# The Paternal Aspect and Relation of the United States Government to Science and the Stand Taken by Secretary Root against the Spread of Tuberculosis in the Western Hemisphere

**Published:** 1906-09

**Authors:** 


### The Paternal Aspect and Relation of the United States Government to Science and the Stand Taken by Secretary Root Against the Spread of Tuberculosis in the Western Hemisphere.

The American government has taken strong ground, in aid of Preventive Legislation in the conflict with tuberculosis.

The American International Congress on Tuberculosis has decided to make a renewal of the assault along the same lines, on which it won its victories at the St. Louis Congress, held at the World's Exposition, in October, 1904.

Hon. Elihu Root brings the splendid sympathetic power of the great republic of the world's civilization in support of the aims and purposes of the American International Congress on Tuberculosis, to be held in the American metropolis, November 14, 15, and 16, 1906.

Mr. Root, on June 5, 1906, six months before the International Congress assembles, advises the Treasurer of the Congress:

"I have to inform you that on May 31st instructions were sent to American diplomatic officers in American States to support the invitation extended by the American International Congress to send delegates to its meeting in November next.

"In compliance with your request, the invitations sent by Mr. Hay concerning the meeting of the Congress, at St. Louis, was embodied in the instructions sent on the 31st ultimo. I enclose a printed copy of them."

The American Secretary of State adds to this announcement the following statement:

"On the same day the American diplomatic representatives in Great Britain, France, Denmark, and the Netherlands, were directed to support the invitation to their respective colonial possessions to be represented at the Congress."

Mr. Root shows great foresight, wisdom and statesmanship in placing the whole moral force of our government behind the great purpose of the American International Congress on Tuberculosis. He has used the same splendidly sympathetic language in bringing it to the official notice of all the governments in the western hemisphere, through our diplomatic corps, that Mr. Secretary Hay employed in recommending the St. Louis Congress to foreign governments.

Mr. Root is making history for both our government and for our people. The language employed is worthy of the cause, worthy of the occasion. Mr. Secretary Root says:

"In instructing the diplomatic officers to give their support to a similar invitation extended by the Congress for their St. Louis meeting, in 1904, my predecessor, Mr. Hay, said:

" 'The humanitarian object which this Congress has in view—to reach by the discussion of scientific men, some result in arresting the spread and averting, so far as it may be found possible, the ravages of this dreadful disease, which now falls with such terrible force and fatality upon the people of the western hemisphere—can not but enlist the sympathy and approval of the government to which you are accredited.

" 'The department will, therefore, be pleased to have you say to that government that this government is in entire sympathy with the work of the proposed Congress, and would be pleased to learn that the government of ———— took a like interest in its success by the acceptance of the committee's invitation and the appointment of three or more scientific gentlemen to represent it at the Congress.

" 'This government would also be pleased if that of.......... could find it convenient to comply with the request of the committee to give the matter publicity, in order that it may come to the knowledge of interested organizations and public spirited citizens of that country.'

"The department will be pleased to have you present the matter of the New York meeting in the same light."

The battle cry of the Congress is Preventive Legislation Against Tuberculosis—To arrest, to avert, to minimize the spread of Consumption, is the battle ground.

The call of the Congress is to the masses of the people, to the men of all professions, the statesman, the publicist, the humanitarian.

It is not a medical question, not confined to medical men, but the call is to all men of all professions, and to the gigantic proportions of the conflict, and the magnitude of the problems, which now confront the health and the safety of that great, that enormous mass of human lives that have been yearly sacrificed to the ravages of this dreadful disease.—*Medico-Legal Journal, N. Y.*

